# Exploring CAR-PBMCs: A Novel Strategy Against EGFR-Positive Tumor Cells

**DOI:** 10.3390/biomedicines13020264

**Published:** 2025-01-22

**Authors:** Alexandru Tîrziu, Oana-Isabella Gavriliuc, Maria-Florina Bojin, Virgil Păunescu

**Affiliations:** 1 Department of Functional Sciences, Immuno-Physiology and Biotechnologies Center, “Victor Babes” University of Medicine and Pharmacy, No. 2 Eftimie Murgu Square, 300041 Timisoara, Romania; alexandru.tirziu@umft.ro (A.T.); florinabojin@umft.ro (M.-F.B.); vpaunescu@umft.ro (V.P.); 2 Center for Gene and Cellular Therapies in the Treatment of Cancer Timisoara-OncoGen, Clinical Emergency County Hospital “Pius Brinzeu” Timisoara, No. 156 Liviu Rebreanu, 300723 Timisoara, Romania

**Keywords:** adoptive cell therapy, chimeric antigen receptor, CAR-T cells, lentiviral transduction, EGFR

## Abstract

**Background:** Chimeric antigen receptor (CAR) T cell therapy has shown significant promise in treating hematological malignancies, yet its application in solid tumors, particularly those expressing the epidermal growth factor receptor (EGFR), remains limited. This study investigates the potential of CAR-engineered peripheral blood mononuclear cells (PBMCs) as a novel adoptive cell therapy against EGFR-positive cancers. **Methods:** Lentiviral transduction at an MOI of 50 was performed to generate specific anti-EGFR second generation CAR-effector cells. The transduced PBMCs were stimulated with cytokines and CD3/CD28 beads to enhance their proliferation and activation. Flow cytometric and real-time cell analysis were performed at various effector-to-target ratios to explore the cytotoxic potential of CAR-PBMCs. **Results:** CAR-PBMCs exhibited improved targeting and cytotoxicity against EGFR-positive cancer cell lines MDA-MB-468 and SK-BR-3, compared to untransduced controls, with unsignificant effects on allogeneic PBMCs. **Conclusion:** CAR-PBMCs hold considerable potential as a therapeutic strategy for EGFR-positive solid tumors, warranting further clinical investigation.

## 1. Introduction

Chimeric antigen receptor (CAR) T cell therapy has emerged as a promising adoptive cell therapy in hematological malignancies [1,2,3]. In the context of solid tumors, CAR-based therapies are intensively studied by targeting surface antigens such as EGFR [4], MUC1, mesothelin [5], GD2 ganglioside [6], EpCAM [7], etc.

Drug resistance and adverse effects are significant challenges in the treatment of cancers targeting the epidermal growth factor receptor (EGFR). One major concern with tyrosine kinase inhibitor (TKI) therapy is the development of drug resistance, as tumor cells can acquire mutations due to genomic instability, leading to a selection pressure that favors resistant clones (the bottleneck effect) [8]. This resistance can manifest in various forms, including the downregulation of target molecules [9] and the emergence of secondary mutations, such as the T790M mutation, which hinders drug binding. Additionally, as tyrosine kinases are involved in numerous signaling pathways, organ toxicity may occur (affecting the lung, heart, skin, gastrointestinal tract, etc.) [10], which can diminish patients’ quality of life. The risk of “on-target-off-tumor” toxicity is another significant limitation, as many tumor cell targets are also present in healthy cells; examples include trastuzumab-induced cardiotoxicity and cetuximab-induced papulopustular rash [11]. Furthermore, the production of autoantibodies against therapeutic antibodies may reduce their effectiveness while increasing the risk of allergic reactions [12].

The epidermal growth factor receptor (EGFR) is a receptor tyrosine kinase that plays critical roles in epithelial cell proliferation, survival, differentiation, and motility by activating multiple signaling pathways including the Ras/MAPK, PI3K/AKT, and PLC/PKC-β signaling cascades [13]. Aberrant activation of the EGFR pathway contributes to the process of tumorigenesis and cancer progression [14]. Therefore, EGFR is considered an important target of multiple cancer therapies, including non-small cell lung carcinoma (NSCLC), head and neck squamous cell cancers (HNSCCs), glioblastoma, and breast, esophageal, colon, colorectal, anal, lung, gastric, bladder, endometrial, melanoma, prostate, pancreatic and ovarian cancers. Several EGFR-targeted therapies have been clinically approved, including tyrosine kinase inhibitors (TKI—erlotinib, gefitinib, icotinib, and afatinib) [15] and monoclonal antibodies (cetuximab, panitumumab, and necitumumab) [4,16].

Recently, chimeric antigen receptor (CAR) T cell therapy has shown promising results against hematologic malignancies by employing the cytotoxic capacity of T cells independent of antigen processing, presentation, and MHC restriction [17]. Consequently, CAR-T cells overcome one of the main mechanisms involved in tumor immune evasion—MHC class I downregulation [18]. Additionally, the intrinsic co-stimulatory capacity of CAR-T cells provides antigen-specific activation, proliferation, survival, and cytotoxicity.

Although multiple preclinical studies provided promising results, the clinical efficacy of anti-EGFR CAR therapy on solid tumors is limited. Therefore, an EGFR-CAR therapy with increased potency is desirable.

Lentiviral transduction is the most frequently used method for generating chimeric antigen receptor-expressing T cells due to its higher transduction efficiency, larger transgene load, and lower immunogenicity compared to other transduction platforms [2]. This method is currently used in clinical manufacturing (Kymriah [19], Breyanzi [20], Abecma [21], Carteyva [22], and Carvykti [23]) against CD19+/ BCMA+ hematologic malignancies (non-Hodgkin’s lymphoma, relapsed or refractory B-cell lymphoblastic leukemia, or relapsed multiple myeloma). The main advantage of this method is the permanent integration of the CAR construct into the host cell genome, providing a persistent expression of the CAR molecule [2,17].

Lentiviruses feature a unique cis-acting element, the central polypurine tract (cPPT), allowing efficient transduction independent of the cell cycle. Their genome includes three Retroviridae genes (gag, pol, env), two regulatory genes (tat, rev), and four accessory genes (vif, vpr, vpu, nef) between long terminal repeats (LTRs). To enhance safety, gag, pol, and rev are spread across three plasmids (pGag/Pol, pRev, pVSV-G). The woodchuck hepatitis virus post-transcriptional regulatory element (WPRE) is added to the 3′-LTR to stabilize transcription products and improve gene expression [17,24].

The core molecular structure of the chimeric antigen receptor comprises an extracellular, a transmembrane and an intracellular domain [25]. The extracellular domain contains the ligand binding domain, with scFv being the most commonly used. scFv is usually derived from the variable heavy and light regions of a monoclonal antibody against a tumor-associated antigen (TAA) such as Her2, EGFR, VEGF, EpCAM, GD2 ganglioside, PD-L1, etc. Selecting the right target antigen is crucial for the effectiveness of CAR-T cell therapy. The ideal target antigen is tumor-specific, enabling the effector cells to distinguish between healthy and tumor cells, limiting “off-target” toxicities [26]. The affinity of the scFv fragment for the target antigen can be modulated to improve specificity and reduce the risk of “on-target/off-tumor” toxicity. Consequently, the CAR downstream signaling, cytokine secretion, cell proliferation, and persistence in vivo will be significantly enhanced [1].

The scFv domain is attached to the transmembrane domain via a hinge region which provides flexibility for the CAR molecule to bind and recognize epitopes with various locations in the protein structure [5]. The hinge regions are typically derived from IgG molecules or native T-cell signaling molecules (CD8a and CD28a) [27]. Compared to Ig-derived hinge domains, CD8a and CD28a hinges allow higher cytokine production capacity (such as interferon-γ and TNF-α) and a greater activation-induced cell death (AICD) potential [28].

The transmembrane domain transduces the extracellular signal into the cytoplasmic milieu. The α-helical structure derived from the CD3 molecule interacts with CD3ζ, which induces T cell activation with subsequent cytotoxicity and cytokine release. As first-generation CARs utilize only the CD3ζ signaling domain as their main signaling motif, their proliferation and persistence in vivo was poor. Co-stimulatory domains such as CD28, 4-1BB, OX40, or ICOS were added in cis with the CD3ζ cytoplasmic domain to overcome this limitation. CD28 provides sustained signaling and enhanced cellular proliferation by augmenting the velocity and intensity of downstream phosphorylation while preventing T-cell exhaustion due to an increase in resistance to CTLA-4-mediated inhibition [29]. 4-1BB enhances immunological synapse formation and provides a greater persistence and differentiation towards central memory, thereby improving clinical efficacy [30]. ICOS and OX40 modulate T-cell differentiation and metabolic activity and inhibit apoptosis and AICD [31,32].

Based on this scaffold, two second-generation CARs with CD28 or 4-1BB (Kymriah and Yescarta), approved by the FDA for clinical use, provided positive results in terms of persistence and efficacy against CD19+ hematologic malignancies [5].

Third-generation CARs utilize both CD28 and 4-1BB co-stimulatory domains, thereby increasing the ZAP70-mediated phosphorylation of signaling proteins, with higher signaling strengths compared to second-generation CARs [33]. Additionally, third-generation CARs provided increased in vivo tumor eradication and upregulation of genes involved in cell migration and adhesion, as well as immune synapse formation.

Although CAR-T cells have been proven effective in treating hematologic malignancies, solid tumors still represent a great challenge for adoptive immunotherapies, mainly due to the characteristics of the tumor microenvironment [34].

The tumor microenvironment is a hostile environment for effector cells due to its high acidity (pH 6.0–6.9), high lactate levels, hypoxia, essential amino acids scarcity, extracellular matrix remodeling, abnormal vasculature, as well as the presence of immunosuppressive proteins and cytokines (such as FasL, IL-10, TGF-β) secreted by the CD4^+^ FOXP3^+^ cells, myeloid-derived suppressor cells, and M2 macrophages [35,36].

This paper investigates an alternative approach to the classical CAR-T cell therapy, by genetically modifying PBMCs using a lentiviral vector encoding a second-generation anti-EGFR CAR. Cells were stimulated using either cytokines (IL-2, IL-15, IL-21) or CD3/CD28 magnetic beads to obtain two heterogenous leukocyte populations NK-polarized and TNK-polarized. To overcome the deleterious effects of the tumor microenvironment, multiple cell types originating from PBMCs were transduced to improve cellular crosstalk and provide resistance to the hostile conditions of TME.

## 2. Materials and Methods

### 2.1. PBMC Isolation

Peripheral blood mononuclear cells (PBMCs) were isolated from five healthy donors from anticoagulant-treated whole blood that was pipetted in a tube over Ficoll-Paque™ PLUS density gradient media (cytiva, Marlborough, MA, USA, cat. # 17144002) and centrifuged for 25 min at 20 °C and 2500 rpm, without brake. The cells were expanded in culture flasks containing XVIVO10 medium (Lonza BioScience, cat. #BP04-743Q, Basel, Switzerland) supplemented with 5% human plasma and 500 U/mL interleukin-2 (Merck, cat. #I2644, Darmstadt, Germany). Two heterogeneous cell populations were obtained (NK-polarized and TNK-polarized) by either cytokine (IL-2, 50 ng/mL, StemCell Technologies, cat. #78145; IL-15, 25 ng/mL, PeproTech EC Ltd., London, UK, cat. #AF-200-15-10UG; and IL-21, 10 ng/mL PeproTech EC Ltd., London, UK, cat. #AF-200-21-10UG) or CD3/CD28 Dynabeads™ stimulation at a bead/cell ratio of 1:1 (Gibco™ BRL, cat. #11131D, Invitrogen, Carlsbad, CA, USA). All peripheral blood samples were collected after informed consent was obtained, following the protocol approved by the Commission of Ethics in Scientific Research at “Victor Babes” University of Medicine and Pharmacy Timisoara, Romania (protocol no. 63/16 October 2024).

### 2.2. Cell Cultures

The MDA-MB-468 (ATCC HTB-132^TM^) cell line was expanded in Dulbecco’s Modified Eagle Medium (DMEM, Sigma-Aldrich, Saint Louis, MO, USA; cat. #D0822), supplemented with 10% Fetal Bovine Serum (FBS, Sigma Aldrich, Saint Louis, MO, USA; #F7524) and 1% Penicillin/Streptomycin solution (Pen/Strep, Sigma Aldrich, Saint Louis, MO, USA; cat. #P4333) at a cell culture density of 3 × 10^4^ cells/cm^2^.

The SK-BR-3 (ATCC HTB-30^TM^) cell line was plated in culture flasks containing the Modified McCoy’s 5a medium (Gibco BRL, cat. #16600082, Invitrogen, Carlsbad, CA, USA) supplemented with 10% fetal calf serum (Sigma Aldrich, cat. #C8056) and 1% Pen/Strep solution at a cell culture density of 3–6 × 10^5^ cells/cm^2^.

K-562 cells (ATCC CCL-243) were seeded in RPMI 1640 medium (Gibco BRL, Invitrogen, Carlsbad, CA, USA; cat. #11875093) supplemented with 10% FBS and 1% Pen/Strep. All cell cultures were incubated at 37 °C, 5% CO_2_.

### 2.3. Target Cell Surface Protein Expression Analysis

EGFR surface expression of the target cell lines SK-BR3 and MDA-MB-468 was analyzed via a flow cytometric assay using APC-conjugated antibodies against EGFR (BioLegend, San Diego, CA, cat. #352906). Additionally, CD1d-expression was assessed using conjugated antibodies anti-CD1d (BD BioSciences, San Jose, CA, USA, cat. #563505). Ligands of activating receptors on the effector cells, such as MICA/B, which might generate a non-CAR-based cytotoxic effect, were also determined using anti-MICA/B-conjugated antibodies (BioLegend, San Diego, CA, USA, cat. #320906). To investigate the potential immune escape response of the target cells, the pro-apoptotic molecule Fas was determined using PE-conjugated anti-Fas antibodies (BioLegend, San Diego, CA, USA, cat. #305608).

### 2.4. The CAR Anti-EGFR Lentiviral Vector

**Figure 1:**
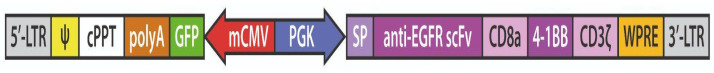
Schematic representation of anti-EGFR CAR lentiviral LentiONE expression vector (GEGTech). The CAR construct contains an anti-EGFR scFv fused to a CD8a hinge and transmembrane domain, a 4-1BB intracellular, and a CD3ζ signaling domain. SP—signaling peptide leader sequence. The CAR is expressed from a PGK promoter, while the GFP (green fluorescent protein) reporter gene is expressed from a miniCMV promoter, part of the same bidirectional promoter unit. 5′,3′-LTR—long tandem repeats; Ψ—RNA packaging signal; cPPT—central polypurine tract; polyA—polyadenylation signal; WPRE—woodchuck hepatitis promoter regulatory element.

Lenti-One™ is a bidirectional third-generation lentiviral vector system encoding a second-generation CAR sequence with a PGK promoter and a GFP reporter from the mCMV promoter. The CAR sequence comprises an scFv derived from the variable heavy and light chains from the monoclonal antibody cetuximab, a CD8a hinge and transmembrane domain, a 4-1BB costimulatory domain, and a CD3ζ intracellular domain for downstream signaling. The Lenti-One™ was supplied by GEGTech as packaged, ready-to-use, lentiviral particles at 10^9^ TU/mL (Figure 1).

### 2.5. Cell Transduction

The NK- and TNK-polarized cell populations were transduced on day 7 of activation with cytokine beads using the spinoculation method combined with polybrene incubation, as this combined method enhanced transduction efficiency compared with the isolated methods alone. Viral transfection was performed at a multiplicity of infection (MOI) of 50, as higher MOI correlates with a higher transduction efficiency, even in hard-to-transfect cells, such as primary NK cells. Cells were centrifuged at 1500 rpm for 5 min, the supernatant was discarded, and the lentiviral particles were suspended in cell culture medium containing Polybrene (Santa Cruz Biotechnologies, Carlsbad, CA, USA, cat. #CAS 28728-55-4), at a concentration of 10 mg/mL. The cell culture plates were centrifuged at 1800× *g* for 1 h, at 32 °C, followed by a 5 h incubation period at 37 °C under 5% CO_2_. The transduction medium was replaced with fresh medium, and the cells were incubated for 72 h at 37 °C under 5% CO_2_.

### 2.6. Transduction Efficiency and CAR Expression

The transduction efficiency was assessed from the expression of GFP using flow cytometry in the overall effector cell population, as well as in the individual cell type populations (T, NK, NKT).

CAR expression assessment was performed by labeling the transduced cells with biotinylated EGFR (R&D Systems, Minneapolis, MN, USA, cat. #BAF231), followed by APC-conjugated streptavidin (BioLegend, San Diego, CA, USA, cat. #405207), according to the manufacturer’s protocol, and subsequent flow cytometric analysis.

### 2.7. In Vitro Toxicity Assays

Direct CAR-mediated toxicity was assessed in an end-point manner by co-culturing the effector cells with calcein violet-stained target cells at different effector-to-target ratios (1:1, 5:1, and 10:1, respectively) for 3 h. Cell viability quantification was performed using flow cytometry by staining the cells using CellTrace™ Calcein Violet, AM (ThermoFischer Scientific, Carlsbad, CA, USA, cat. #C34858) and propidium iodide (BD Biosciences, San Jose, CA, USA, cat. #51-66211E).

Effector-mediated cell death was also assessed by continuous real-time cell analysis (RTCA) using the xCELLigence RTCA DP system (Agilent Technologies, Santa Clara, CA, USA). MDA-MB-468 and SK-BR3 cells were seeded on 16-well E-plates 16 PET (cat. #30-060-0890) at a density of 5000 cells/well and cultured for 24 h before co-culturing them with CAR-expressing cells and untransduced cells at 10:1 effector/target ratio. The target cells alone were used as a negative control. Cell viability was evaluated using the dynamics of the cell index over a 24 h time interval (xCELLigence RTCA Software Pro 1.2).

Cytolysis was also assessed by evaluating the formation of large structures, named clumps, by the GFP-positive effector cells and cell death by the detachment of target cells (DiD-stained, ThermoFischer Scientific, Carlsbad, CA, USA, cat. #V22887) from the bottom of the wells.

### 2.8. Statistical Analysis

Statistical analysis and data visualization were performed using GraphPad Prism version 8.0.2. For continuous variables, Student’s *t*-test and ANOVA were conducted, where applicable. For proportions, the χ^2^-test was performed. The values were expressed as the mean (M) ± standard error of mean (SEM). The null hypothesis was rejected for a *p* value less than 0.05. The flow cytometry data and plots were analyzed using BD FACSVerse version 1.0.6.

## 3. Results

### 3.1. Cell Expansion Analysis

Compared to NK cell-polarized cells, cell expansion of the TNK group was significantly better, expressing a 10-fold increase in the cell number. In the TNK-polarized cells, from days 0 to 28, the percentage of dead cells decreased, and this can be attributed to the stimulatory effect of the CD3/CD28 beads (15 ± 2% on day 0 vs. 2 ± 0.8% at day 28, *p* = 0.0019). Additionally, the proportion of NK cells significantly decreased (25 ± 8% at day 0 vs. 0% at day 28, *p* = 0.005), while the percentage of NKT cells became significantly representative (19 ± 5% at day 0 vs. 46 ± 7% at day 28, *p* = 0.005). However, the genetic modification of the cells (CAR-positive vs. CAR-negative) did not affect the changes in the distribution of T cells (CD3 + CD56−), NKT cells (CD3 + CD56+), and NK cells (CD3-CD56+) induced by in vitro culture (T cells: 91 ± 8% vs. 89 ± 6%, *p* = 0.74; NKT cells: 3 ± 1% vs. 7 ± 3%, *p* = 0.15).

**Figure 2:**
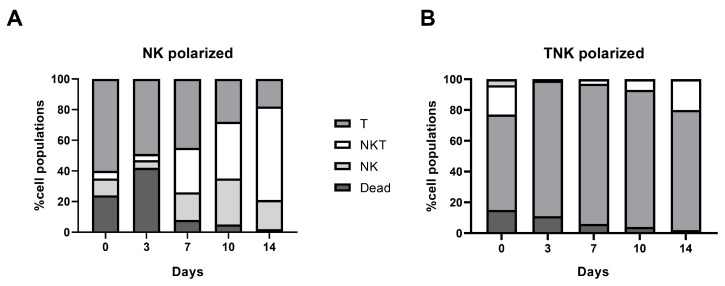
Cell expansion analysis for NK-polarized (**A**) and TNK-polarized cells (**B**). Compared to Day 0, at day 14, a marked increase in the NKT cell population was observed in the NK-polarized group. In the TNK-polarized group, a marked increase in both T and NKT populations was noted, with a significant decrease in the NK cell population. No statistically significant differences were observed in the cell populations before (day 7) and at 72 h post-transduction.

In the NK-polarized cells, the percentage of dead cells decreased significantly from day 0 to day 14 (23 ± 6% vs. 2 ± 0.5%, *p* < 0.001), the proportion of NKT and NK cells increased significantly (5 ± 1% vs. 60 ± 8%, *p* < 0.001; 10 ± 1% vs. 18 ± 5%, *p* < 0.001), while the T cell population decreased (61 ± 9% vs. 18 ± 6%, *p* < 0.001). The analysis of cell proportions before and after transduction (at 72 h) revealed no significant changes in the dead and NKT cell populations (7 ± 5% vs. 4 ± 1%, *p* = 0.302; 29 ± 3% vs. 36 ± 6%, *p* = 0.241), but a significant increase in NK cells (18 ± 2% vs. 33 ± 6%, *p* = 0.005) with a simultaneous T cell decrease (41 ± 3% vs. 27 ± 3%, *p* = 0.002) (Figure 2).

### 3.2. Transduction Efficiency Analysis

Surface expression of EGFR-specific CAR on transduced cells ranged from 55 to 76% of all GFP-positive cells, with T cells expressing the CAR at higher intensities, followed by NKT, and a very low to almost undetectable expression on NK cells.

**Figure 3:**
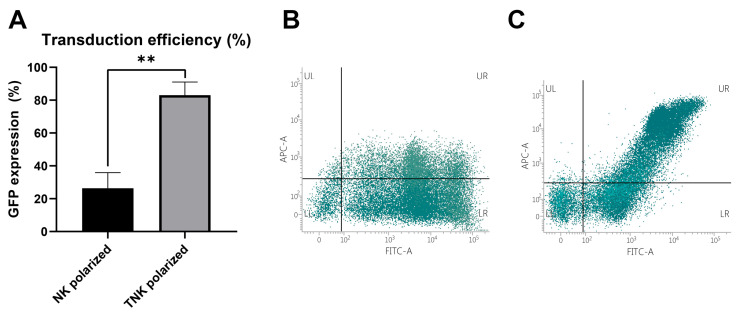
(**A**) Transduction efficiency assessed by the flow cytometry analysis of GFP-expressing cells at 3 days post-transduction. In the TNK-polarized group (stimulated with cytokines and beads), a higher transduction efficiency was observed (83 ± 8% vs. 26 ± 9%, *p* = 0.001). (**B**,**C**) Dot plot graphs of CAR-NK-polarized cells (**B**) and CAR-TNK-polarized cells (**C**); cells were treated with biotinylated EGFR and streptavidin-APC. The upper right quadrant (UR) indicates the population of CAR+ cells (FITC-positive and APC-positive); **—*p* < 0.01.

CAR expression was better preserved in the TNK-polarized cells compared to the NK-polarized cells (83 ± 8% vs. 26 ± 9%, *p* = 0.001) (Figure 3), with the culture evolving into a 70:30 mixed phenotype of NKT and T cells by week 4 (day 28).

Considering the enhanced proliferation and transduction efficiency of the TNK-polarized cells, this culture was further employed to assess its cytotoxic effects on EGFR-expressing tumor cells.

### 3.3. Target Cell Surface Protein Expression Analysis

**Figure 4:**
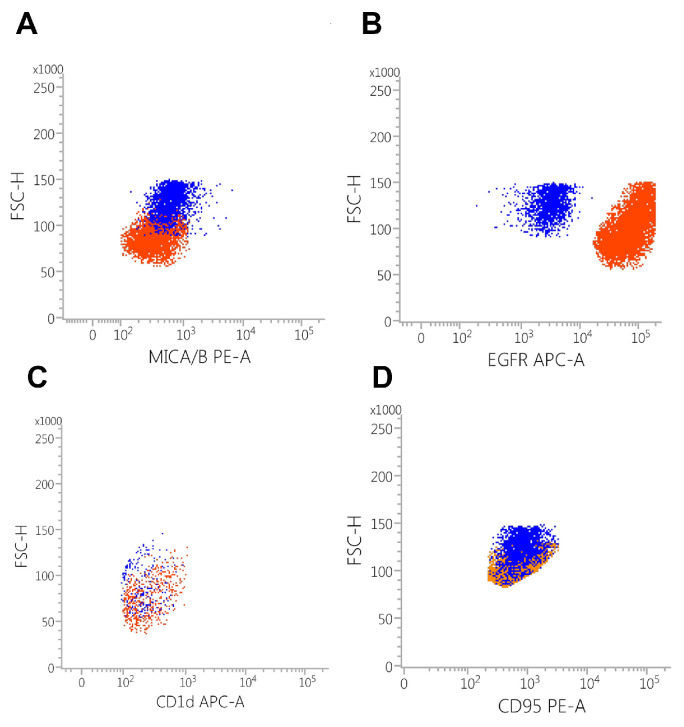
(**A**) Dot-plot analysis of MICA/B expression in MDA-MB468 (red) cells vs. SK-BR3 (blue) cells showed a similar expression profile. (**B**) The MDA-MB468 cell line (red) exhibited a higher EGFR expression compared to SK-BR3 cells (blue). (**C**) CD1d expression in both cell types is reduced, but with a slight increase in the MDA-MB468 cell line. (**D**) CD95 (Fas) expression in MDA-MB468 cells (red) vs. SK-BR3 (blue) exhibited a similar expression profile in both cell types.

The analysis of target cell surface proteins revealed a significantly higher expression of EGFR in MDA-MB468 cells compared to SK-BR3 cells, with values of 84.30% and 82.92%, respectively (*p* = 0.987). Both cell lines exhibited a robust expression profile for the pro-apoptotic molecule Fas, with MDA-MB468 showing 78.35% and SK-BR3 at 70.72% (*p* = 0.931). Notably, CD1d expression was slightly elevated in the MDA-MB468 line (13.73%) compared to SK-BR3 (9.31%, *p* = 0.905). Regarding non-specific effector cell activation, the MICA/B markers demonstrated increased expression in both cell lines, with MDA-MB468 at 62.9% and SK-BR3 at 28.65% (*p* = 0.665). Detailed dot-plots illustrating cell surface protein expressions are presented in Figure 4.

When evaluating mean fluorescence intensity (MFI), MDA-MB468 cells exhibited higher levels of EGFR compared to SK-BR3, reaching statistical significance (MDA-MB468: 89,596 ± 562.51 mean fluorescence units (MFU) vs. SK-BR3: 3707.50 ± 19.59 MFU, *p* = 0.02). Interestingly, the non-specific activator molecule MICA/B showed increased expression in the SK-BR3 line (545.5 ± 117.16 MFU vs. 493 ± 46.54 MFU, *p* = 0.83). Moreover, CD1d was overexpressed in MDA-MB468 cells (951 ± 55.52 MFU vs. 362.5 ± 19.18 MFU, *p* = 0.098). The expression levels of Fas were comparable between the two lines (1568.33 ± 796.49 MFU vs. 1060.33 ± 72.07 MFU, *p* = 0.5598).

### 3.4. Cytotoxicity Assay

The cytotoxic activity of CAR-PBMCs was assessed via flow cytometry by incubating the target cells (SK-BR3 and MDSA-MB468) with the effector cells for 3 h. The cell co-cultures were further stained using Calcein Violet AM and propidium iodide to evaluate the number of target cells, along with the percentage of dead cells.

**Figure 5:**
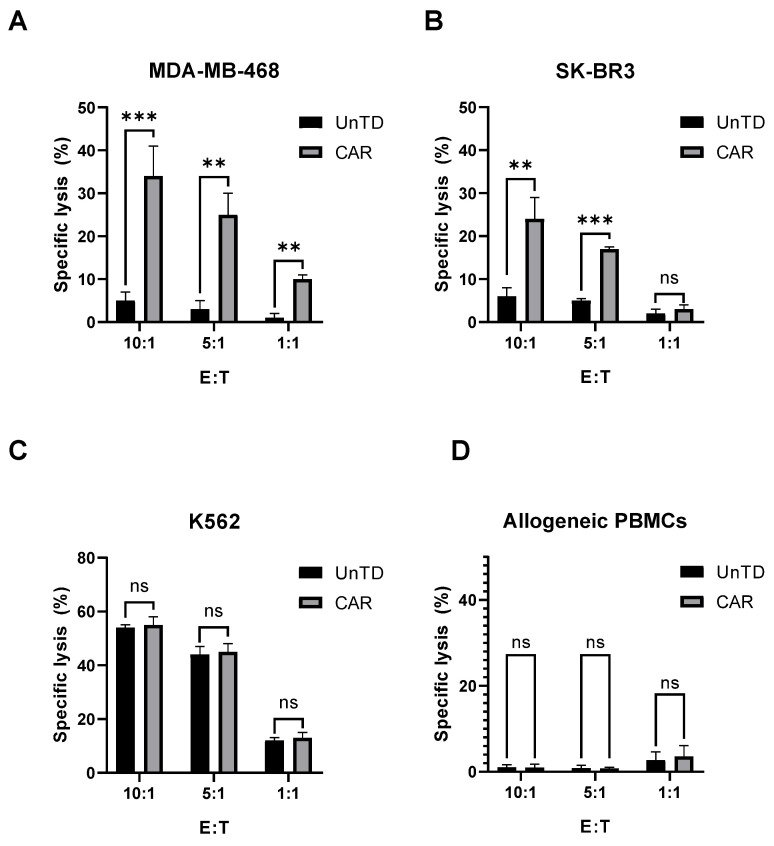
Short-term cytotoxic activity of anti-EGFR CAR transduced cells compared to untransduced cells against calcein-labeled target MDA-MB-468 cells (**A**), SK-BR3 cells (**B**), K562 cells (**C**), and allogeneic PBMCs (**D**). The transduction significantly increased the effector cells’ cytotoxic activity, which correlated with EGFR expression levels in EGFR-expressing tumor cell lines MDA-MB-468 (E:T = 10:1—34 ± 4% vs. 5 ± 1%, *p* < 0.001; E:T = 5:1—25 ± 2% vs. 3 ± 1%, *p* < 0.05; E:T = 1:1—10 ± 0.5% vs. 1 ± 0.5%, *p* < 0.05) and SK-BR-3 (E:T = 10:1—24 ± 2% vs. 6 ± 1%, *p* < 0.05; E:T = 5:1—17 ± 0.2% vs. 5 ± 0.2%, *p* < 0.001; E:T = 1:1—3 ± 1% vs. 2 ± 1%, *p* = 0.28), but without statistical significance in EGFR-negative, NK-sensitive K562 cells, nor allogeneic PBMCs (E:T = 1:1—3.58 ± 2.53% vs. 2.74 ± 1.90%, *p* = 0.66; E:T = 5:1—0.79 ± 0.26% vs. 0.84 ± 0.62%, *p* = 0.90; E:T = 10:1—0.99 ± 0.79% vs. 1.06 ± 0.53%, *p* = 0.90); ns—not significant (*p* > 0.05); **—*p* < 0.01; *** *p* < 0.001.

**Figure 6:**
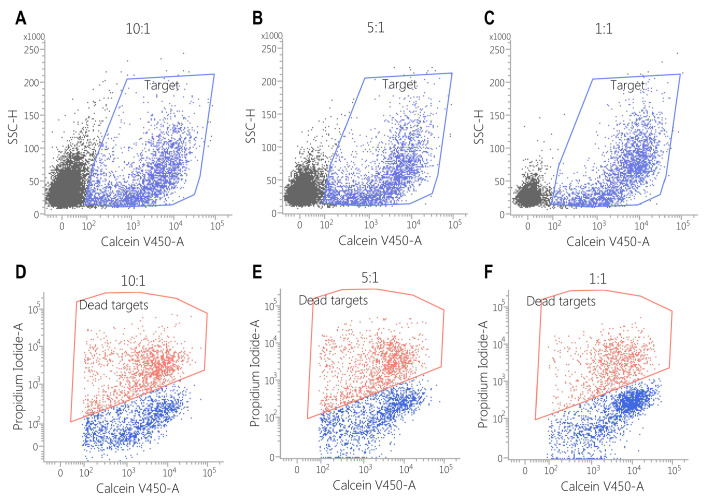
Flow cytometric assessment of cytotoxicity of CAR-PBMCs against the MDA-MB468 cell line. The cells were stained with calcein violet (blue polygon gate, plots (**A**–**C**)), while the cytotoxic effect was explored by propidium iodide staining (red polygon gate, plots (**D**–**F**)). The cytotoxic effect correlates with the number of effector cells. (E:T = 10:1—34 ± 4.04% E:T = 5:1—25 ± 2.78% E:T = 1:1—10 ± 0.57%).

**Figure 7:**
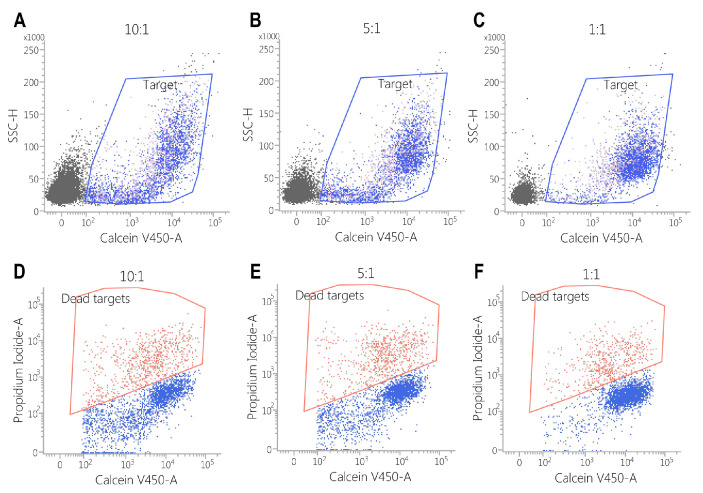
Flow cytometric assessment of the cytotoxicity of untransduced PBMCs against the MDA-MB468 cell line. Cells were stained with calcein violet to identify the target cells (blue polygon gate in plots (**A**–**C**)) and propidium iodide to assess cytotoxic effects (red polygon gate in plots (**D**–**F**)). The results demonstrate that cytotoxicity correlates with the effector-to-target (E:T) ratio, with observed percentages of cell death as follows: E:T = 10:1: 5 ± 0.57%; E:T = 5:1: 3 ± 1.15%; E:T = 1:1: 2 ± 0.52%.

Against the MDA-MB-468 cell line, CAR-EGFR-positive cells expressed an enhanced cytotoxic activity compared to untransduced cells, even at an effector/target ratio of 1:1 (E:T = 10:1—34 ± 4% vs. 5 ± 1%, *p* < 0.001; E:T = 5:1—25 ± 2% vs. 3 ± 1%, *p* < 0.05; E:T = 1:1—10 ± 0.5% vs. 1 ± 0.5%, *p* < 0.05) (Figure 5, Figure 6 and Figure 7).

**Figure 8:**
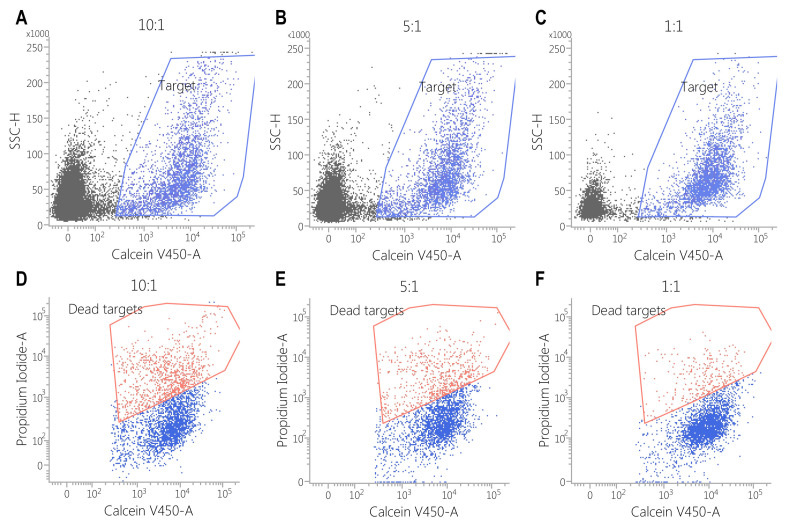
Flow cytometric assessment of the cytotoxicity of CAR-PBMCs against the SK-BR3 cell line. Target cells were stained with calcein violet (blue polygon gate in plots (**A**–**C**)) to identify viable cells, while propidium iodide staining (red polygon gate in plots (**D**–**F**)) was used to evaluate cytotoxic effects. The results indicate that cytotoxicity is positively correlated with the effector-to-target (E:T) ratio, with the following observed percentages of cell death: E:T = 10:1: 24 ± 2.88%; E:T = 5:1: 17 ± 0.28%; E:T = 1:1: 3 ± 0.43%.

**Figure 9:**
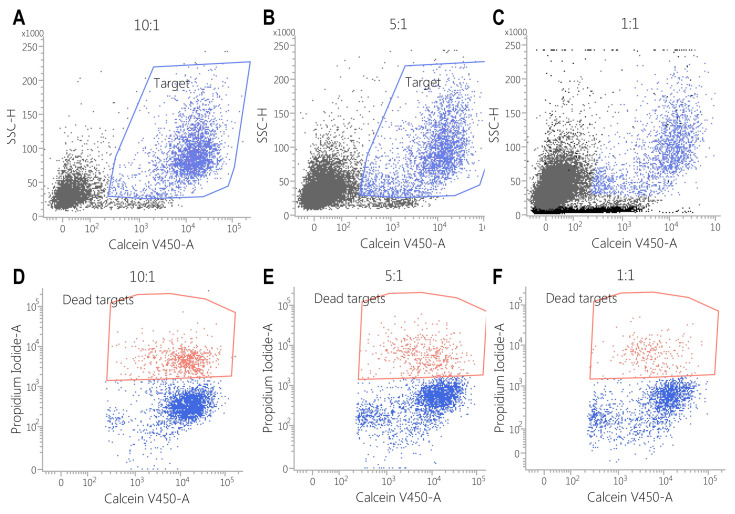
Flow cytometric assessment of cytotoxicity of untransduced PBMCs against the SK-BR3 cell line. Target cells were stained with calcein violet (blue polygon gate in plots (**A**–**C**)), while propidium iodide staining (red polygon gate in plots (**D**–**F**)) was used to evaluate cytotoxic effects. The results indicate a correlation between cytotoxicity and the effector-to-target (E:T) ratio, with the following observed percentages of cell death: E:T = 10:1: 6 ± 1.15%; E:T = 5:1: 5 ± 0.28%; E:T = 1:1: 2 ± 0.57%.

**Figure 10:**
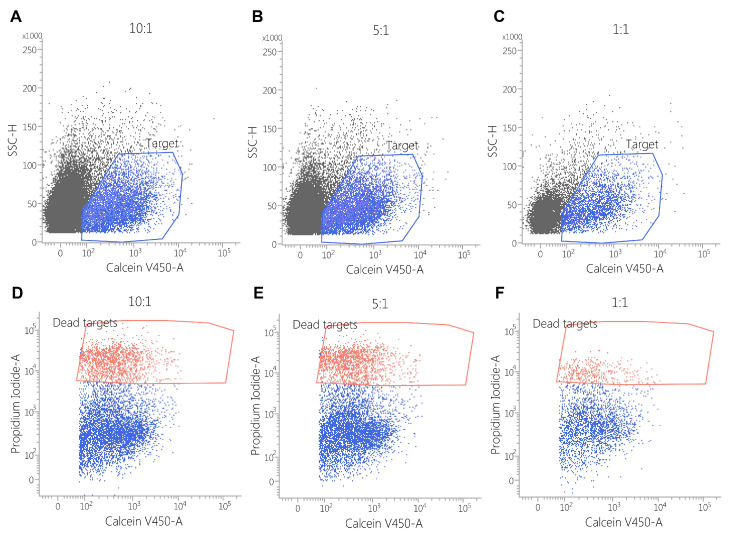
Flow cytometric analysis of CAR-PBMC cytotoxicity against the K562 cell line. Target cells were stained with calcein violet (blue polygon gate in plots (**A**–**C**)), while propidium iodide staining (red polygon gate in plots (**D**–**F**)) assessed cytotoxic effects. Results demonstrate a correlation between cytotoxicity and the effector-to-target (E:T) ratio, with the following percentages of cell death observed: E:T = 10:1—54 ± 1%; E:T = 5:1—44 ± 3%; E:T = 1:1—12 ± 1%.

**Figure 11:**
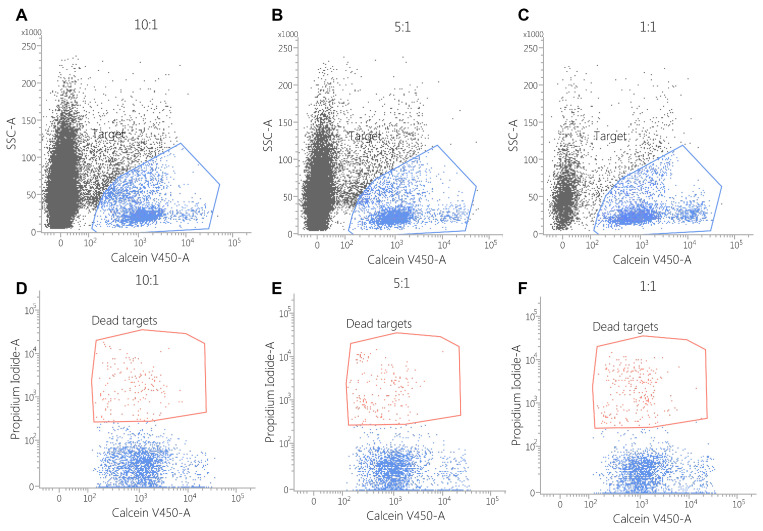
Flow cytometric assessment of cytotoxicity of CAR-PBMCs against allogeneic PBMCs. Target cells were stained with calcein violet (blue polygon gate in plots (**A**–**C**)), while propidium iodide staining (red polygon gate in plots (**D**–**F**)) was used to evaluate cytotoxic effects. The results indicate unsignificant cytotoxicity even at an effector/target ratio of 10:1: E:T = 1:1—3.58 ± 2.53% E:T = 5:1—0.79 ± 0.26%; E:T = 10:1—0.99 ± 0.79%.

This effect was also observed against SK-BR3 cells, but without significant differences in the E:T of 1:1 (E:T = 10:1—24 ± 2% vs. 6 ± 1%, *p* < 0.05; E:T = 5:1—17 ± 0.2% vs. 5 ± 0.2%, *p* < 0.001; E:T = 1:1—3 ± 1% vs. 2 ± 1%, *p* = 0.28) (Figure 5, Figure 6, Figure 7, Figure 8 and Figure 9). As K562 cells do not constitutively express EGFR, and we did not observe any statistically significant differences between the CAR-transduced cells and the control group (E:T = 10:1—54 ± 1% vs. 55 ± 3%, *p* = 0.61; E:T = 5:1—44 ± 3% vs. 45 ± 3, *p* = 0.704; E:T = 1:1—12 ± 1% vs. 13 ± 2%, *p* = 0.48) (Figure 5 and Figure 10). Cytotoxicity against SK-BR-3, which express lower levels of EGFR, was decreased (24.6% for SK-BR-3 vs. 34.5% for MDA-MB-468, *p* = 0.11) at an effector/target ratio of 10:1, but still significantly improved over untransduced cells (Figure 6 and Figure 8). Genetically modified cells did not display any significant cytotoxic effect against allogeneic PBMCs, even at an effector/target ratio of 10:1 (E:T = 1:1—3.58 ± 2.53% vs. 2.74 ± 1.90%, *p* = 0.66; E:T = 5:1—0.79 ± 0.26% vs. 0.84 ± 0.62%, *p* = 0.90; E:T = 10:1—0.99 ± 0.79% vs. 1.06 ± 0.53%, *p* = 0.90) (Figure 5 and Figure 11).

**Figure 12:**
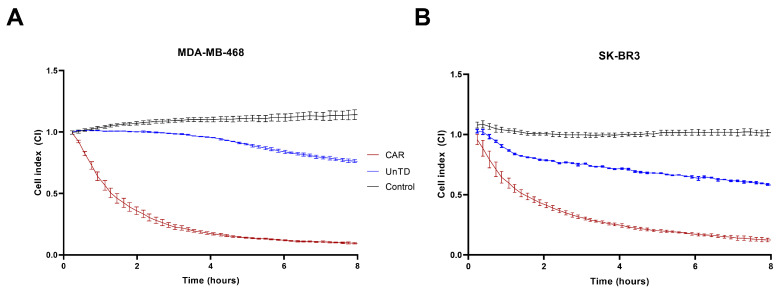
(**A**) RTCA analysis of EGFR-expressing MDA-MB468 cells incubated for 24 h with transduced anti-EGFR CAR cells (red) and untransduced cells (blue). (**B**) RTCA analysis of the EGFR positive SK-BR3 cell line, incubated for 24 hours with CAR-expressing cells (red) and untransduced PBMCs (blue). In the first 2 h, a rapid decline in cell index was observed in both cell lines treated with CAR cells (red). However, this effect was more pronounced in the MDA-MB-468 group. A poor decline was also observed in the untransduced group (blue), which may account for non-specific lysis. The control group experienced constant proliferation (black).

**Figure 13:**
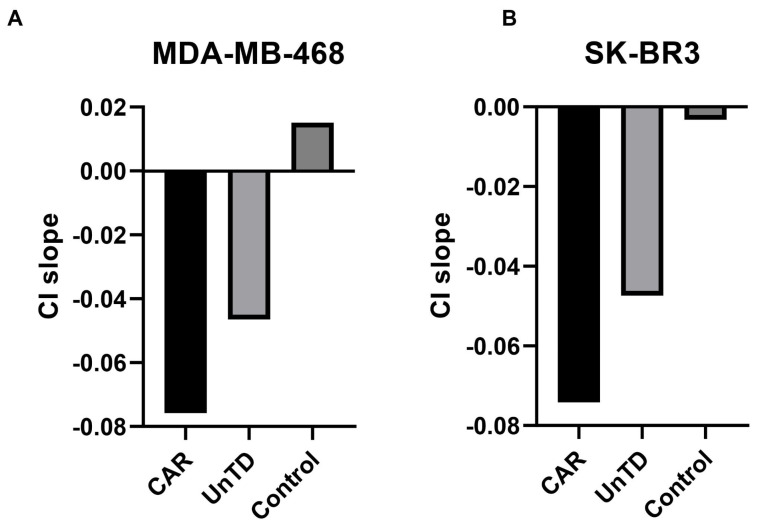
(**A**) Cell index slopes for MDA-MB468 cells incubated with CAR-expressing and untransduced cells (UnTD). (**B**) Cell index slopes for SK-BR3 cells treated with CAR-expressing cells and untransduced (UnTD) cells. The slopes of the cell indices (CI slope) were more negative in the CAR-treated target cells compared to untransduced cells. In the MDA-MB-468 control cells (**A**), the slope had an overall positive value, suggestive for cell proliferation, compared to a negative value in the SK-BR3 control group, which may reflect the differences in EGFR expression as a mechanism for cell survival, as well as an increased expression of the non-specific activator molecule MICA/B in SK-BR3. The CI slopes for tumor cells incubated with CAR-PBMCs were comparable, with net negative values (MDA-MB468: −0.076 vs. SK-BR3: −0.074, *p* = 0.1714).

**Figure 14:**
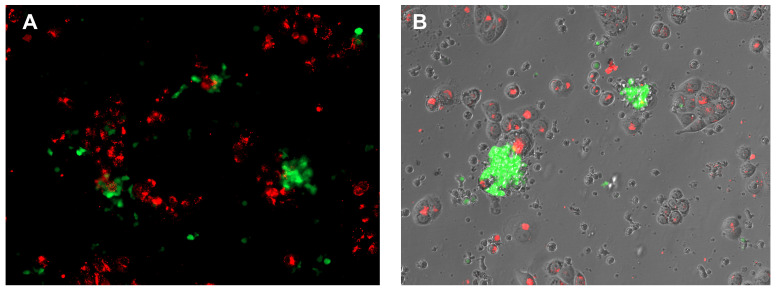
Fluorescence microscopy (**A**) and overlay fluorescence-transillumination (**B**) image showing activated CAR-TNK cells (green) and MDA-MB468 cells stained red with DiO. The green-stained CAR-TNK cells exhibit clumping behavior, indicating their activation and interaction with the MDA-MB468 target cells. This visualization highlights the dynamic interactions between CAR-TNK and tumor cells in the microenvironment.

After the addition of effector cells to their target, an immediate decline in the CI was observed, with more than 50% cytolysis occurring in the first 2 h for CAR-transduced cells, while untransduced cells showed significantly less cell death. Complete cytolysis of tumor targets was achieved in 8 h from the addition of effector cells. Target cells alone displayed minimal cell death and continued to survive until the end of the monitoring period. Analysis of the dynamics of the cytolysis indicated that the cytotoxic activity of CAR cells was more pronounced in the case of the MDA-MB-468 cell line, compared to SK-BR-3, which correlates with higher expression of the CAR target EGFR. Target cell killing by untransduced cells can be explained by unspecific activation of effectors via NK- or T-cell receptors (NKp30, NKp46, NKG2D, FcR). However, it was considerably lower than specific killing induced by recognition of EGFR by CAR (Figure 12, Figure 13 and Figure 14).

## 4. Discussion

This current study investigated the immunotherapeutic potential of CAR anti-EGFR-transduced PBMCs, generated using the LentiOne™ EGFR lentiviral vector developed by GEGTech™. Our cellular study model consisted of a mixed phenotype of T, NK, and NKT cells isolated from five healthy volunteers. The target cells consisted of two breast cancer cellular lines, MDA-MB468 and SK-BR3, which were analyzed in terms of differential target molecule (EGFR) expression, as well as the presence of non-specific (MICA/B) and specific (CD1d) activating and inhibitory molecules (Fas).

MDA-MB468 cells exhibited a significantly higher expression of EGFR compared to SK-BR3 cells, suggesting that MDA-MB468 may have a greater reliance on EGFR signaling pathways, potentially influencing their behavior in therapeutic contexts. Both cell lines displayed high levels of Fas expression, although MDA-MB468 showed slightly lower levels than SK-BR3. This finding may reflect differences in apoptotic signaling pathways and could have implications in the context of solid tumor-directed immunotherapy. Although CD1d expression in both cell lines was poor, a slightly increased expression of CD1d in MDA-MB468 cells compared to SK-BR3 may suggest a potential role in modulating immune responses, possibly affecting their interactions with NKT cells. The expression of MICA/B markers was comparable in both tumor cells lines, highlighting the supplementary role provided by non-specific activation.

The isolated PBMCs were subjected to two distinct methods of cell stimulation and expansion: one involving cytokines (IL-2, IL-15, IL-21) and the other utilizing CD3/CD28-Dynabeads™. After 14 days of stimulation, the resulting populations displayed different cellular compositions. Nonetheless, a shared characteristic of both the TNK and NK-polarized populations was a notable increase in the production of NKT cells.

The advantages of using mixed cellular platforms (such as CAR-PBMCs) include the capacity of transducing a diverse population of immune effectors, as well as characterizing simultaneously several types of effector cells in terms of cellular viability, cellular crosstalk, and cytotoxic capacity, as these models better reflect the in vivo diversity. This approach could also allow for a non-MHC-restricted recognition of diverse tumor targets through either the CAR NK-cell activating receptors or endogenous TCR receptors of NKT cells triggered by glycolipids presented via CD1d, improving the effector cells’ resistance to exhaustion. By combining CAR-expressing NK, NKT, and T cells, the cytotoxic effects will increase, as tumor cells that downregulate their MHC class I molecules will also be recognized, with a potent cytolytic activity sustained by diverse modes of killing. Compared to CAR-T cells that exert their cytotoxic functions by both chimeric antigen and T cell receptors, CAR-NKT cells promote indirect anti-tumoral effects by stimulating dendritic cell maturation and activation of tumor-infiltrating CD8+ lymphocytes and NK cells via TNF-α and IFN-γ release. Furthermore, CAR-NKT cells also recognize tumor antigens in an MHC-I-and-II-independent manner, reducing the risk of graft-versus-host disease. CAR-NKT cells can leverage both CAR-dependent and invariant T cell receptor (iTCR)-dependent mechanisms for tumor recognition. This dual action allows them to target tumor-associated macrophages (TAMs) and myeloid-derived suppressor cells (MDSCs), enhancing their antitumor efficacy [37]. The unique properties of mixed CAR-NK and CAR-NKT cells make them excellent candidates for combination therapies with other treatment modalities, such as immune checkpoint inhibitors or conventional therapies. Their ability to activate other immune components further enhances their therapeutic potential against tumors [38].

The use of PBMCs as the starting material can streamline the manufacturing process by potentially eliminating the need for prior T cell separation. This simplification could result in more efficient production protocols, reducing both time and costs while ensuring high viability and functionality of the final product. Such efficiency is particularly critical in the context of aggressive cancers, where timely intervention can significantly impact patient outcomes.

The differential composition between the two cell populations was also investigated by Watkinson et al., who also reported a modification of cell type composition in PBMCs following different cytokine stimulation regimens (IL-2, IL-15) [39].

Low numbers and impaired function of circulating NKT cells in cancer patients is associated with a poor prognosis, emphasizing their role in antitumoral immunity [40].

Direct tumor toxicity, promoted by the recognition of CD1d-presented lipid antigens, is mediated by perforins, granzyme B, and over-expression of the Fas ligand. Although many solid tumors are associated with CD1d downregulation as a mechanism of immune escape, CAR-mediated recognition of the tumor-associated antigen restores their function in the tumor microenvironment [41]. Through pro-inflammatory cytokine release (TNF-α, IFN-γ), CAR-NKT cells have the potential to reshape the immune activity inside the tumor microenvironment by promoting dendritic cell proliferation via CD40-CD40L interaction and antigen presentation with subsequent activation of CD4+ and CD8+ tumor-infiltrating lymphocytes. CAR-NKT cells can also promote anti-tumor immunity by stimulating IL-12 release by dendritic cells, leading to NK cell stimulation and IFN-γ production. Furthermore, NKT cells can also induce the phenotypic transformation of M2 macrophages into the pro-inflammatory M1 phenotype [40,42].

The LentiOne™ anti-EGFR vector comprises a PGK promoter, providing an efficient expression of CAR molecules in T and NKT cells. However, the CAR expression in NK cells was poor, as these cells are widely known as “hard-to-transfect” cells. This finding confirms what was already published in the literature and suggests that other promoters such as EF-1a or CMV may perform better on this cellular type. Additionally, other non-viral transduction platforms must be explored (CRISPR, mRNA electroporation, or transposon-mediated integration) [43].

The addition of 4-1BB co-stimulatory domain was also explored by Heczey et al. on CAR-NKT cells, where phenotypic changes to a Th1-like response in both T and NKT cells occurred, with increased γ-interferon and GM-CSF release and decreased levels of the anti-inflammatory cytokines IL-4 and IL-10 [42].

Lentiviral transduction was not associated with increased cell cytotoxicity nor changes in cell type composition between days 0 and 3 post-transduction, suggesting that the third-generation lentiviral delivery system is safe and well-tolerated by the effector cells. NK-polarized cells showed a decrease in CAR expression over time, while the phenotype of transduced cells became mostly CD56+, with only NKT cells still expressing the CAR. This finding may be attributed to the intrinsic anti-viral effect of NK cells, which hamper the process of lentiviral transduction [44]. The memory phenotype of transduced cells in culture became predominantly effector memory, with the naive cells subgroup restricted to a small population of T cells. NKT cells differentiated from T cells also demonstrated a tendency for the downregulation of co-receptors CD8 and CD4. It was shown that CD4-negative variants of NKT cells may exhibit increased antitumor immunity compared to their CD4+ counterparts [41].

CAR-transduced cells had demonstrated an increased anti-EGFR specific cytotoxic response against the EGFR-expressing MDA-MB-468 and SK-BR3 cell lines both in flow cytometric and real-time cell analyses (RTCA). We observed an increased cytotoxic activity in the MDA-MB-468 group compared to the SK-BR3 cell line. This finding may be attributed to the higher expression of EGFR on the surface of these cells compared to SK-BR3. The cytotoxic effect began early in the co-culturing process, as the cell index decreased by approximately 50% in the first 2 h in both target cell lines.

CAR-EGFR positive cells showed significantly enhanced cytotoxic activity against the MDA-MB-468 cell line compared to untransduced cells, even at a low effector-to-target (E:T) ratio of 1:1. This indicates the effectiveness of CAR-EGFR modification in targeting and killing high-EGFR-expressing tumor cells. Moreover, CAR-EGFR-positive cells also exhibited cytotoxicity against SK-BR3 cells. However, the differences in cytotoxicity at an E:T ratio of 1:1 were not statistically significant. This suggests that SK-BR3 cells, which express lower levels of EGFR, may not be as effectively targeted by CAR-EGFR-modified cells as MDA-MB-468 cells. The observed decrease in cytotoxicity against SK-BR3 cells (24.6% vs. 34.5% for MDA-MB-468 at an E:T ratio of 10:1) further emphasizes the relationship between EGFR expression levels and the effectiveness of CAR-T/NK/NKT cell-mediated lysis.

The lack of significant differences in cytotoxicity against K562 cells, which do not express EGFR, can be mainly attributed to the classical interactions between NK and K562 cells, reinforcing the specificity of CAR-EGFR positive cells for EGFR-expressing targets. This specificity is crucial for minimizing off-target effects in potential therapeutic applications.

Notably, given the potential clinical use of anti-EGFR CAR cells, the TNK cells showed no cytotoxicity against allogeneic PBMCs, which supports the claims of reduced GvHD for this cellular platform.

In the RTCA analysis, the addition of CAR-transduced effector cells to MDA-MB-468 targets resulted in a rapid decline in cell viability, with over 50% cytolysis occurring within the first 2 h. This highlights the effectiveness and prompt action of CAR-EGFR modified T cells against high-EGFR expressing tumors. Additionally, complete cytolysis of MDA-MB-468 cells was achieved within 8 h, demonstrating the potent antitumor activity of CAR-transduced cells. In contrast, untransduced cells exhibited significantly less cytotoxicity, underscoring the specificity and enhanced functionality provided by CAR modification. The cytotoxicity observed with untransduced cells can be attributed to non-specific activation through various NK or T-cell receptors (e.g., NKp30, NKp46, NKG2D). However, this non-specific killing was significantly lower than that mediated by CAR recognition of EGFR, emphasizing the enhanced specificity and efficacy of CAR-modified T cells.

Target cells cultured without effector cells showed minimal cell death throughout the monitoring period, indicating that the observed cytotoxic effects were specifically due to the action of CAR-transduced effector cells rather than inherent susceptibility of the target cells.

The cytotoxic effects observed using calcein violet AM/ PI flow cytometry and RTCA analysis can be attributed to cellular cross-talk and cytokine release. EGFR-positive tumor cells are recognized by CAR-T cells, leading to perforin and granzyme-mediated cell lysis. In addition, target cells present lipid antigens via CD1d, which along with EGFR recognition by CAR, will activate CAR-NKT cells, leading to increased production of inflammatory cytokines such as IFN-γ, TNF-α, and IL-2, further enhancing effector cell proliferation and cytotoxic capacity. CAR-NK-specific lysis is mediated by CAR-EGFR interactions, along with non-specific MICA/B-NKG2D activation. An advantage of recruiting CAR-NKT and CAR-NK cells is an additional bypass of the classic HLA class I-TCR interaction, which was found to be suppressed in tumor cells as a mechanism of immune escape. Tumor cell destruction generated cell fragments which will be phagocytosed by the dendritic cells, leading to increased antigen presentation with stimulating effects on CAR-T cells and TILs and increased IL-12 and IFN-γ production.

Although CAR-T cell therapy has been proven successful in treating hematologic malignancies, solid tumors face several challenges. One typical characteristic of solid tumors is the dynamic composition of surface antigens, which has a significant impact on CAR adoptive cell therapy, since CAR-T cells have been proven ineffective against cells with low antigen density [45]. This issue can be further resolved using bi-specific CARs (such as tandem CARs) so that multiple antigens can be recognized by a single effector immune cell [17].

The ideal target antigen for CAR-T cells must be tumor-specific and play a crucial role in tumor growth and development [46]. As the majority of CAR-T cell targets are tumor-associated antigens (molecules that are overexpressed in malignant tissues, but also present on healthy cells) the rate of on-target/off-tumor toxicities is still increased [47]. In a trial of Her2-targeted CAR-T cell therapy for metastatic colon cancer, a patient underwent respiratory failure due to CAR-T-mediated cytotoxic activity against Her2-expressing alveolar cells [48]. Additionally, a carbonic anhydrase IX-targeted CAR-T cell therapy against renal cell carcinoma produced liver toxicity due to co-expression of the target antigen in the bile duct epithelium [49]. This type of toxicity can be overcome by either using other effector cells, such as NKTs, by employing new-generation CARs (logic-gate control CARs with OR gate—tandem and dual CARs—or AND logic-gates—split-CARs or synNotch CARs), or by making use of the viral oncolytic therapy [3,17,50].

CAR-T cell therapy may be associated with clinical toxicities such as the cytokine-release syndrome or neurotoxicity due to organ damage induced by the massive release of pro-inflammatory cytokines (IL-6, IL-1, IFN-γ, TNF-α) [51]. Several solutions to this problem have been proposed, such as inducible CARs, CAR-T cells with suicide genes, AND-gate CARs, or by using CAR-NK or CAR-NKT cells [43].

Solid tumors generate physical barriers by the highly organized extracellular matrix due to increased lysyl-oxidase activity, along with specific glycoprotein expression. CAR-T cells produced using peripheral blood mononuclear cells exhibit inadequate chemotactic activity when recruited in the tumor stroma compared to TILs. During the process of in vitro expansion and repetitive stimulation, along with the high concentrations of inhibitory cytokines in the tumor microenvironment, CAR-T cells develop an exhausted phenotype (upregulation of PD-1, LAG3, TIM3, and TIGIT and decreased secretion of IL-2, TNF-α, and IFN-γ). However, the effect of CAR-PBMCs, which comprise a heterogenous population of transduced immune effector cells, may disrupt the physico-chemical barriers imposed by the tumor microenvironment through cytokine release that will augment the effect of cytotoxic lymphocytes (CAR-T, CAR-NK, and TILs).

Although the in vitro results are promising, this study has several limitations. Notably, the absence of in vivo data using murine tumor models limits the validation of the in vitro findings. Future studies incorporating in vivo experiments in murine models are essential to confirm and extend the outcomes observed in vitro. Furthermore, the study did not evaluate the cytokine release profile of the CAR-based platform. Future research is necessary to thoroughly characterize these aspects and enhance our understanding of the platform’s potential.

## 5. Conclusions

Considering the favorable results regarding cell transduction and the cytotoxic effects on EGFR+ tumor cells, this study presents a promising approach to CAR-based platforms through the transduction of PBMCs. This strategy integrates the effects of CAR-T, CAR-NK, and CAR-NKT cells, resulting in a more heterogeneous population of CAR-effector cells that may effectively overcome tumor immune escape mechanisms. This diversity enables CAR-PBMCs to harness a broader range of immune effector mechanisms.

## Data Availability

The original contributions presented in this study are included in the article. Further inquiries can be directed to the corresponding author.
